# Sequential release of TNFα and phospholipase A_2_ in a rat model of LPS-induced pleurisy

**DOI:** 10.1080/09629359791703

**Published:** 1997-06

**Authors:** C. Cicala, M. Bucci, F. D′Acquisto, L. Parente, G. Cirino

**Affiliations:** 1 Department of Experimental Pharmacology University of Naples ]Federico II' Via D. Montesano 49 Naples 80131 Italy; 2 Institute of Pharmacology and Pharmacognosy University of Palermo Via Forlanini 1 Palermo 90134 Italy

## Abstract

The levels of extracellular phospholipase A_2_ (sPLA_2_) and TNFα, and cell accumulation were measured in the pleural washings obtained at different times following the induction of *Escherichia coli* lipopolysaccharide (LPS, 100 μg/cavity) pleurisy in rats. TNFα peaked at 2 hours (3036 ± 160.3 units/ml) and decreased thereafter. Conversely, levels of sPLA_2_ peaked at 48 hours (1.97 ± 0.64 ng/ml) and were increased further (14.02 ± 4.16 ng/ml) by pretreatment with anti-TNFα antibody. Cell accumulation was not affected by antibody pretreatment. These data indicate that the sPLA_2_ enzyme is involved in LPS-induced pleurisy. The enzyme seems not to be stimulated by TNFα which may be involved in the downregulation of sPLA_2_ in this model of inflammation.


Mediators of Inflammation, 6, 211–215 (1997)

### Sequential release of TNFα and phospholipase $A_2$ in a rat model of LPS-induced pleurisy

C. Cicala[1,CA], M. Bucci[1], F. D'Acquisto[1], L. Parente[2], G. Cirino[1]

[1]Department of Experimental Pharmacology, University of Naples 'Federico II' Via D. Montesano 49, 80131, Naples, Italy; and [2]Institute of Pharmacology and Pharmacognosy, University of Palermo, Via Forlanini 1, 90134 Palermo, Italy

[CA]Corresponding author
Tel: (+39) 81 7486443
Fax: (+39) 81 7486403

**The levels of extracellular phospholipase $A_2$ ($sPLA_2$) and TNFα, and cell accumulation were measured in the pleural washings obtained at different times following the induction of *Escherichia coli* lipopolysaccharide (LPS, 100 μg/cavity) pleurisy in rats. TNFα peaked at 2 hours (3036 ± 160·3 units/ml) and decreased thereafter. Conversely, levels of $sPLA_2$ peaked at 48 hours (1·97 ± 0·64 ng/ml) and were increased further (14·02 ± 4·16 ng/ml) by pretreatment with anti-TNFα antibody. Cell accumulation was not affected by antibody pretreatment. These data indicate that the $sPLA_2$ enzyme is involved in LPS-induced pleurisy. The enzyme seems not to be stimulated by TNFα which may be involved in the down-regulation of $sPLA_2$ in this model of inflammation.**

**Key words:** Endotoxin, Phospholipase $A_2$, Pleurisy, Rat, Tumour necrosis factor α

### Introduction

The extracellular form of phospholipase $A_2$ has been shown to be involved in several inflammatory disorders, including those arising from Gram negative bacterial infections.[1,2]

Phospholipase $A_2$ ($PLA_2$) activation is a rate limiting step in the generation of arachidonic acid, the substrate necessary for eicosanoid generation. The secreted form of $PLA_2$ ($sPLA_2$ group II) has been found at high levels in the serum and locally during inflammation in experimental animals and humans,[3] and there is *in vitro* evidence that its induction is under cytokine control.[4–7] Furthermore, $sPLA_2$ has been proposed to play a role in septic shock. To date, however, the relationship between cytokines and $sPLA_2$ levels in inflammatory exudates and in the serum is still controversial.[2,8] It has been shown that intrapleural injection of LPS into rats elicits an inflammatory response characterized by leucocyte accumulation.[9] Furthermore, challenge of isolated guinea pig lung with endotoxin generates TNFα[11] and the presence of TNFα in the bronchoalveolar lavage fluid of experimental animals has been demonstrated following intratracheal administration of LPS.[12] It appears that tumour necrosis factor alpha (TNFα) is a cytokine playing a pivotal role in the development of endotoxin-induced inflammation.[10]

We have examined whether intrathoracic injection of bacterial endotoxin into rats is capable of causing the release of $sPLA_2$ and TNFα, and the potential relationship between this enzyme and this cytokine in this model of inflammation.

### Materials and Methods

### Pleurisy

Pleurisy was induced in enflurane anaesthetized male Wistar rats (250–300 g; Charles River, Milan, Italy) by intrathoracic injection of *E. coli* lipopolysaccharide (LPS, 0127:B8; 100 μg/cavity) in a final volume of 200 μl. Control animals received an equivalent volume of phosphate buffered saline (PBS). Different groups of animals were treated with a polyclonal anti-TNFα antibody (3 mg/kg i.v.; TAB, London) or control IgG (3 mg/kg i.v.) immediately before the induction of pleurisy. Animals were killed with $CO_2$ at 2, 6, 24, 48, or 96 hours following the injection of LPS or PBS. Groups of animals pretreated with anti-TNFα antibody, or control IgG, were killed after 2 and 48 hours, the time required to obtain peak TNFα and $sPLA_2$ levels respectively. Immediately after sacrifice the thoracic cavity was opened and washed with 2 ml of heparinized (5 UI/ml) sterile saline. The fluid volume was collected in graduated tubes and centrifuged at 125 × *g* for 10–15 min. The

© 1997 Rapid Science Publishers pellet was suspended in 1 ml of PBS and total leucocyte count was evaluated. Supernatant was further centrifuged at 650 × *g* for 10–15 min, immediately frozen and kept at –20°C until assayed for TNFα and $sPLA_2$.

### Leucocyte count

Total leucocyte count was evaluated by optical microscopy in the cell suspension diluted with Turk's solution. Differential cell count was performed by counting air dried smears stained with Giemsa reagent under an oil immersion objective. Counts are reported as number of cells $\times 10^6$/cavity.

### TNFα determination

TNFα in the cell-free pleural washing was assessed on WEHI-164 cells by a bioassay using recombinant human TNFα (Sigma) as the reference standard, and rabbit anti-murine TNFα antiserum (Genzyme) which cross reacts with rat TNFα to assess the specificity of TNFα dependent cytolitic activity.[13] TNFα is expressed as units of TNFα/ml, where one unit is defined as the quantity required to lyse 50% of WEHI-164 cells.

### $PLA_2$ assay

A sandwich assay for $sPLA_2$ was run according to the method described by Santos *et al.*[8] with some modifications. Plates were coated overnight with a monoclonal anti-human recombinant $sPLA_2$ antibody (BA11), 10 μg/ml, in 50 mM sodium bicarbonate buffer, pH 9.6. Six cycles of washing with 0.1% w/v Tween-20 in PBS were done at each step and all incubations were at 4°C. The plates were blocked overnight with 10 mg/ml of gelatin in PBS. Human recombinant $sPLA_2$ was diluted in 50 nM Tris-HCl, pH 7.5, 0.1% w/v Tween-20, 1% v/v foetal calf serum (FCS), to obtain the desired dilution, and 0.05 ml were added to the wells at 4°C for 1 hour (low temperature decreases non-specific $PLA_2$ binding). Bound human recombinant $sPLA_2$ was detected by incubation for 90 min with 0.05 ml of a 1:2000 dilution of rabbit anti-human recombinant $sPLA_2$ (n.207) in assay buffer. Plates were washed and 0.1 ml of a solution containing 1 mg/ml of tetramethylbenzidine in DMSO, mixed with 10 ml of acetate buffer (0.1 M, pH 5.0) and 0.125 ml of diluted hydrogen peroxide (0.2 ml 30% hydrogen peroxide and 7 ml of water), were added to each well. After 15–20 min, 0.025 ml of stop solution ($H_2SO_4$, 2 M) was added and the yellow colour read at 450 nm by an ELISA reader. $sPLA_2$ in the cell-free pleural washing was expressed as ng/ml.

### Histological analysis

Lungs from control or LPS-injected animals were removed and fixed in neutral buffered formalin before being embedded in paraffin. Sections (4 μm) were stained with hematoxylin and eosin.

### Statistical analysis

Data are expressed as mean ± SEM and analysed with a computerized package for statistical comparison.[14] To compare groups of animals, unpaired two tailed Student's t test was used. Data on TNFα were analysed by the Mann–Whitney test. A $P < 0{\cdot}05$ was taken as significant.

### Materials

Recombinant human $sPLA_2$ produced in mammalian cells, monoclonal anti-human recombinant $sPLA_2$ antibody (BA11) and rabbit polyclonal anti-human recombinant $sPLA_2$ antibody (n. 217) were a generous gift from Dr J. L. Browning (Biogen). Enflurane was purchased from Abbott (Italy). Lipopolysaccharide from *E. coli* (serotype 0127:B8), PBS and heparin were purchased from Sigma (Italy). Antibody to human TNFα was a generous gift from TAB London, and has been shown to neutralize rat TNFα *in vitro* (TAB London, unpublished data).

### Results

### Leucocytes

Following intrathoracic injection of LPS, total leucocyte count was significantly increased only at 48 and 96 hours, while at 6 hours there was a significant reduction in the leucocyte count compared to control value. Polymorphonuclear count was significantly increased at 24 and 48 hours, significantly reduced at 6 hours and returned to control levels after 96 hours. Monocyte count was significantly increased at 96 hours, and significantly reduced below control levels at 6 and 24 hours (Fig. 1).

### TNFα determination

TNFα in the cell-free pleural washing peaked at 2 hours following LPS injection. At the successive time points, TNFα levels were reduced progressively, becoming undetectable at 96 hours (Fig. 2).

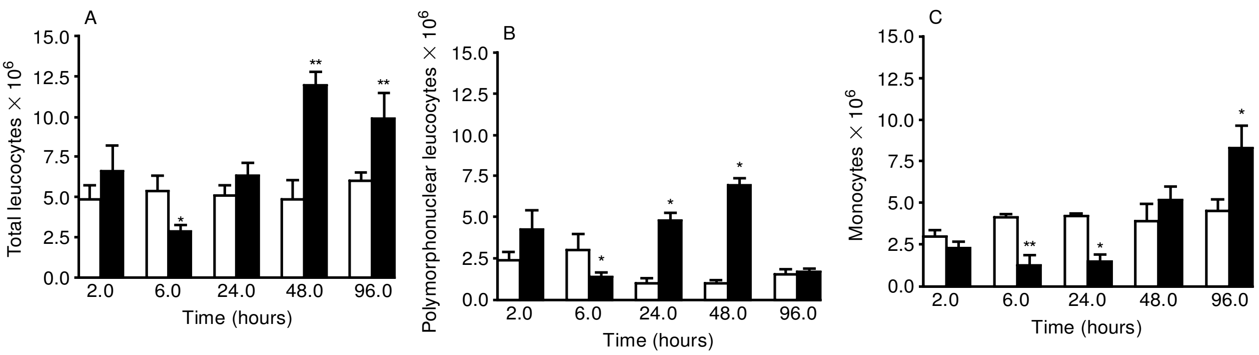

FIG. 1. Time course of cell accumulation in the pleural space following LPS (100 μg/cavity) injection: (A) total leucocytes; (B) polymorphonuclear leucocytes; (C) monocytes. Control, open bars; LPS, filled bars. $^{*}P<0.05$; $^{**}P<0.01$. Student's t-test, n = 4–6.

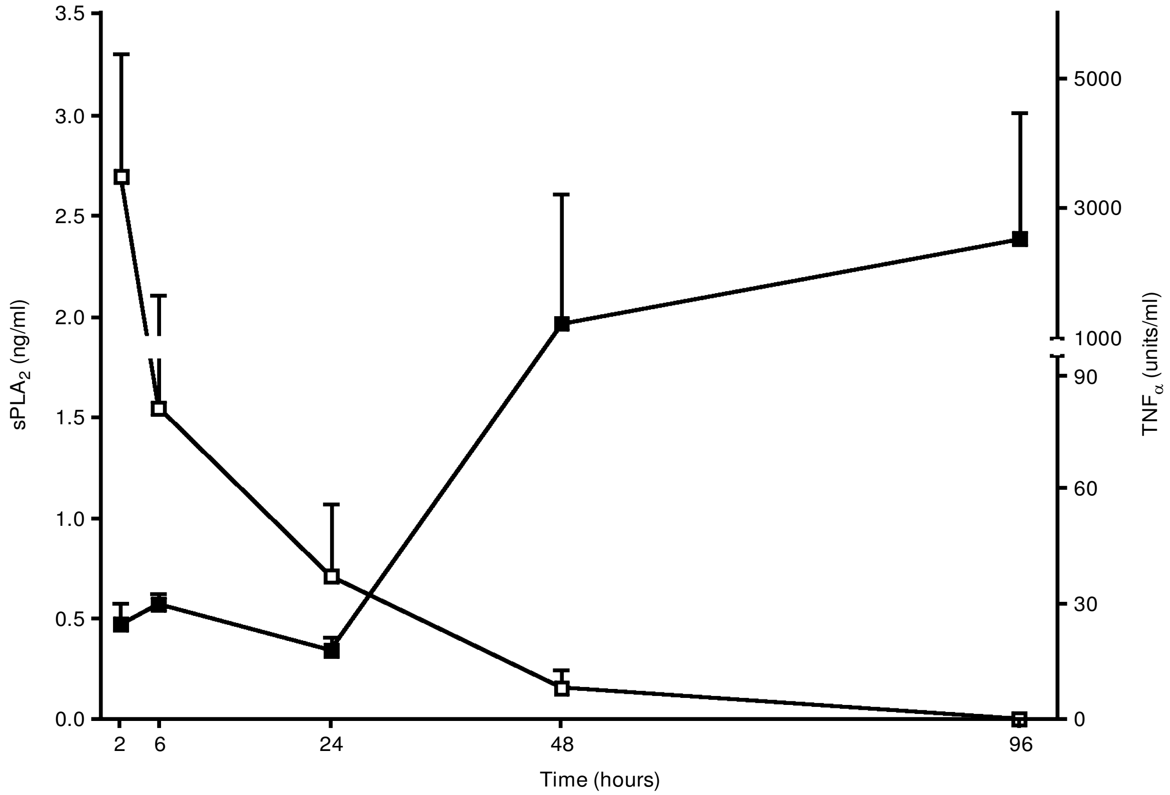

FIG. 2. Time course of $sPLA_2$ (■; n = 4–6) and TNFα (□; n = 3–7) levels in the cell-free pleural washing following LPS (100 μg/cavity) administration.

### $PLA_2$ determination

Phospholipase $A_2$ levels in the cell-free pleural washing were detectable at 2 hours, reached their maximum at 48 hours and were stable up to 96 hours (Fig. 2).

### Pretreatment with anti-TNFα antibody

Levels of TNFα at 2 hours were significantly reduced by anti-TNFα antibody (118·4 ± 41·4 units/ml vs 3036 ± 1603 units/ml as determined by the Mann–Whitney test, $P<0{\cdot}01$, n = 5). Pretreatment with anti-TNFα antibody significantly increased $sPLA_2$ levels at 48 hours (Fig. 3). Both total and differential cell analysis were unaffected by antibody pretreatment (data not shown).

### Histological analysis

In the histological sections of lungs obtained from either PBS or LPS injected animals there was neither leukocyte infiltration, nor alteration in the cell lining of tissue.

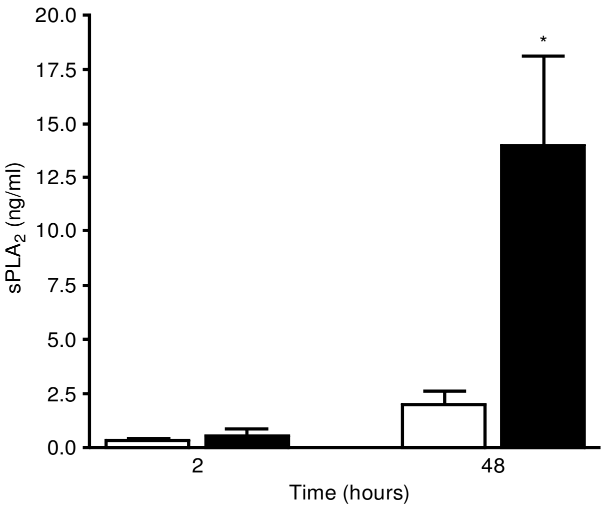

FIG. 3. Effect of anti-TNFα antibody (3 mg/kg i.v.) on $sPLA_2$ levels at 2 and 48 hours following LPS (100 μg/cavity) administration (n = 4). Control, open bars; treated, filled bars. * $P < 0.05$. Student's t-test.

### Discussion

By using a highly specific immunoenzymatic assay, we have demonstrated the presence of extracellular $sPLA_2$ group II in the cell-free pleural washing at different time points following LPS injection, providing direct evidence for a role of this enzyme in the pleurisy triggered by LPS. In our model, the appearance in the cell-free pleural washing of increased levels of $sPLA_2$ was delayed compared to TNFα levels. Indeed, $sPLA_2$ increased in the cell-free pleural washing only when TNFα levels started to decline, and it reached its maximum at 48 hours, when TNFα levels were very low. At the same time, cells significantly accumulated: polymorphonuclear leucocytes reached their maximum at 48 hours, and at 96 hours the pleural washing consisted almost totally of monocytes. When animals were pretreated with anti-TNFα antibody, TNFα levels were significantly reduced. However, cell accumulation did not parallel the increase in TNFα and pretreatment with anti-TNFα antibody did not affect total and differential cell count, suggesting that resident pulmonary cells, stimulated by LPS, may account for TNFα production and that TNFα is not involved in LPS-induced leucocyte margination in the pleural space. These data are consistent with data reported by others, who demonstrated that IL-1 and not TNFα is mostly responsible for LPS-induced neutrophil migration to an inflammatory site.[15] Moreover, it is known that adhesion molecules contribute greatly to LPS-induced leucocyte margination in the lung and their expression is under the control of multiple cytokines.[12] Other authors report that the ability of TNFα inhibitors to protect animals from flogogen-induced lung injury is not linked to a reduction in PMN sequestration.[16] In the light of this knowledge, it is not unexpected that the inhibition of TNFα alone did not affect leucocyte accumulation.

Surprisingly, when we pretreated animals with the anti-TNFα antibody, we observed a strong increase in $sPLA_2$ levels in the cell-free pleural washing at 48 hours. Thus, it seems that TNFα does not exercise a positive control on $sPLA_2$ induction *in vivo*, rather its inhibition causes $sPLA_2$ levels to increase further, at least in this experimental model of pleurisy. Systemic administration of the antibody allows us to rule out the hypothesis of any interference of the antibody with ELISA assay reagents. Although there is much evidence that *in vitro* cytokines are the major inducers of $sPLA_2$ synthesis and secretion from several different cell types, there is no direct evidence that *in vivo* $sPLA_2$ secretion is under cytokine control. A positive correlation has been found between TNFα and $sPLA_2$ plasma levels both in human and in experimental animals challenged with bacterial endotoxin, as well as an increase in plasma $sPLA_2$ levels following TNFα injection in volunteers,[1,2] but this evidence has never been followed by clarification of the *in vivo* role of the enzyme. In 1993 synthesis and secretion of a 14 kDa $sPLA_2$ from guinea pig alveolar macrophages was demonstrated and it was found that the enzyme was not involved in the release of arachidonic acid metabolites.[17] More recently the same research group has shown that expression of $sPLA_2$ in guinea pig alveolar macrophages is downregulated by an inflammatory stimulus such as FMLP,[18] and it has been found that the release of arachidonic acid metabolites from guinea pig alveolar macrophages *in vivo* is reduced below control levels 24 hours after pretreatment with LPS.[19] Up to now, the role of $sPLA_2$ *in vivo* has been unresolved; following these more recent findings, it has been suggested that the secreted form of $PLA_2$ might have a protective role at site of injury.[17–21]

It is noteworthy that 6 hours following LPS administration there was a significant reduction in leucocyte number in the pleural space, paralleled by a reduction in both granulocyte and monocyte counts. Although there was neutrophil accumulation in the lung at 24 hours, the total leucocyte count did not increase since the monocyte count was still reduced below the control level. This reduction could be explained by an increased cell adhesivity which precedes cell accumulation. Indeed, recent data have demonstrated that injection of increasing doses of IL-1 or TNFα in to the pleural space of rats causes a bell-shaped cell accumulation curve. This finding has been shown to be linked to the increased cell adhesivity, evoked by high doses of IL-1 and TNFα.[22]

In conclusion, our data demonstrate that following intrapleural injection of endotoxin into rats, TNFα and $sPLA_2$ group II appear sequentially in the pleural washings. The early appearance of TNFα is in agreement with data reported by others who describe TNFα as an early response cytokine. The high levels of $sPLA_2$ in the late phase of the inflammation further suggest a role for the extracellular form of the enzyme in inflammation triggered by bacterial endotoxins. TNFα is probably produced by resident pulmonary cells and does not seem to be directly involved in cell accumulation. TNFα does not stimulate the enzyme induction, rather there is evidence that its inhibition increases $sPLA_2$ levels, suggesting that this cytokine could participate in a mechanism of downregulation of the enzyme during inflammation; however, this hypothesis requires further clarification.

### References

1. Vadas P, Hay JB. Involvement of circulating phospholipase $A_2$ in the pathogenesis of the hemodynamic changes in endotoxin shock. *Can J Physiol Pharmacol* 1983; **61**: 561–566.
2. Pruzanski W, Wilmore DW, Suffredini A, *et al.* Hyperphospholipasemia $A_2$ in human volunteers challenged with intravenous endotoxin. *Inflammation* 1992; **16**: 561–570.
3. Vadas PJ, Browning J, Edelson J, Pruzanski W. Extracellular phospholipase $A_2$ expression and inflammation: the relationship with associated disease states. *J Lipid Med* 1993; **8**: 1–3.
4. Kerr JS, Stevens TM, Davis GL, Mc Laughlin JA, Harris RR. Effects of recombinant interleukin-1 beta on phospholipase $A_2$ activity, phospholipase $A_2$ mRNA levels and eicosanoid formation in rabbit chondrocytes. *Biochem Biophys Res Comm* 1989; **165**: 1079–1084.
5. Nakazato Y, Simonson MS, Herman WH, Konieczkowski M, Sedor JR. Interleukin-1 alpha stimulates prostaglandin biosynthesis in serum-activated mesangial cells by induction of a non-pancreatic (type II) phospholipase $A_2$. *J Biol Chem* 1991; **266**: 14119–14127.
6. Shalkwijk C, Pfeilschifter J, Marki F, Van Den Bosch H. Interleukin-1 beta, tumor necrosis factor and forskolin stimulate the synthesis and secretion of group II phospholipase $A_2$ in rat mesangial cells. *Biochem Biophys Res Comm* 1991; **74**: 268–275.
7. Pfeischifter J, Shalkwijk C, Briner AV, Van Den Bosch H. Cytokine-stimulated secretion of group II phospolipase $A_2$ by rat mesangial cells. *J Clin Invest* 1993; **92**: 2516–2523.
8. Santos AA, Browning JL, Scheltinga MR, *et al.* Are events after endotoxemia related to circulating phospholipase $A_2$? *Ann Surg* 1994; **219**: 183–192.
9. Bozza TP, Castro-Faria-Neto CH, Martins MA, *et al.* Pharmacological modulation of lipopolysaccharide-induced pleural eosinophilia in the rat; role for a new generated protein. *Eur J Pharmacol* 1993; **248**: 41–47.
10. Tracey KJ, Beutler B, Lowry SF, *et al.* Shock and tissue injury induced by recombinant human cachectin. *Science* 1986; **234**: 470–473.
11. Horgan MF, Palace GP, Everitt JE, Malik AB. TNF-α release in endotoxemia contributes to neutrophil-dependent pulmonary edema. *Am J Physiol* 1993; **264**: H1161–1165.
12. Tang WW, Yi ES, Remick DG, *et al.* Intratracheal injection of endotoxin and cytokines. IX. Contribution of CD11a/ICAM-1 to neutrophil emigration. *Am J Physiol* 1995; **269**: L653–659.
13. Garrelds IM, Zijlstra FJ, Tak CJAM, Bonta IL, Beckman I, Ben Efreim S. A comparison between two methods for measuring necrosis factor in biological fluids. *Agents Actions* 1993; **38**: C90–91.
14. Tallarida RJ, Murray RB. *Manual of pharmacological calculations with computer programs*. London: Springer Verlag, 1987.
15. Cybulsky MI, McComb DJ, Movat HZ. Neutrophil leukocyte emigration induced by endotoxin. Mediator roles of interleukin-1 and tumor necrosis factor alpha 1. *J Immunol* 1988; **140**(9): 3144–3149.
16. Andres DW, Kutkoski GJ, Quinlan WM, Doyle NA, Doerschuk CM. Effect of pentoxifylline on changes in neutrophil sequestration and emigration in the lungs. *Am J Physiol* 1995; **268**: L27–32.
17. Hidi R, Vargaftig BB, Touqui L. Increased synthesis and secretion of a 14-kDa phospholipase $A_2$ by guinea pig alveolar macrophages. *J Immunol* 1993; **151**: 5613–5623.
18. Vial D, Senorale-Pose M, Havet N, Molio L, Vargaftig BB, Touqui L. Expression of the type II phospholipase $A_2$ in alveolar macrophages. Downregulation by an inflammatory signal. *J Biol Chem* 1995; **270**: 17327–17332.
19. De Castro CMM, Bureau MF, Nahori MA, Dumarey CH, Vargaftig BB, Bachelet M. Modulation by dexamethasone of phospholipase $A_2$ activities in endotoxemic guinea pigs. *J Appl Physiol* 1995; **79** (84): 1271–1277.
20. Weinrauch Y, Elsbach P, Madsen LM, Foreman A, Weiss J. The potent anti-*Staphyloccus aureus* activity of a sterile rabbit inflammatory fluid is due to a 14 kDa phospholipase $A_2$. *J Clin Inv* 1996; **97**: 250–257.
21. Hack EC, Wolbink GJ, Schalkwijk C, Speijer H, Hermens W, van den Bosch H. A role for secretory phospholipase $A_2$ and C-reactive protein in the removal of injured cells. *Immunol Today* 1997; **18**: 111–115.
22. Utsunomiya I, Ito M, Watanabe K, Tsurufuji S, Matsushima K, Oh-Shi S. Infiltration of neutrophil by intrapleural injection of tumour necrosis factor, interleukin-1, and interleukin-8 in rats, and its modification by actonomycin D. *Br J Pharmacol* 1996; **117**: 611–614.

ACKNOWLEDGEMENTS. We are indebted with Dr D. C. Smith (PhD) for his generous gift of the anti-TNFα antibody and for his critical revision of the manuscript.

**Received 25 March 1997;
accepted in revised form 14 May 1997**
